# A nomogram for predicting the risk of pulmonary embolism in neurology department suspected PE patients: A 10-year retrospective analysis

**DOI:** 10.3389/fneur.2023.1139598

**Published:** 2023-04-05

**Authors:** Qiang Jianling, Jin Lulu, Qiu Liuyi, Feng Lanfang, Ma Xu, Li Wenchen, Wang Maofeng

**Affiliations:** ^1^Department of Biomedical Sciences Laboratory, Affiliated Dongyang Hospital of Wenzhou Medical University, Dongyang, Zhejiang, China; ^2^Department of Pathology, Affiliated Dongyang Hospital of Wenzhou Medical University, Dongyang, Zhejiang, China; ^3^Department of Respiratory, Affiliated Dongyang Hospital of Wenzhou Medical University, Dongyang, Zhejiang, China; ^4^Department of Vascular Surgery, Affiliated Dongyang Hospital of Wenzhou Medical University, Dongyang, Zhejiang, China; ^5^Department of Neurology, Affiliated Dongyang Hospital of Wenzhou Medical University, Dongyang, Zhejiang, China

**Keywords:** pulmonary embolism, neurology department, retrospective analysis, risk assessment models, nomogram

## Abstract

**Objective:**

The purpose of this retrospective study was to establish a numerical model for predicting the risk of pulmonary embolism (PE) in neurology department patients.

**Methods:**

A total of 1,578 subjects with suspected PE at the neurology department from January 2012 to December 2021 were considered for enrollment in our retrospective study. The patients were randomly divided into the training cohort and the validation cohort in the ratio of 7:3. The least absolute shrinkage and selection operator regression were used to select the optimal predictive features. Multivariate logistic regression was used to establish the numerical model, and this model was visualized by a nomogram. The model performance was assessed and validated by discrimination, calibration, and clinical utility.

**Results:**

Our predictive model indicated that eight variables, namely, age, pulse, systolic pressure, hemoglobin, neutrophil count, low-density lipoprotein, D-dimer, and partial pressure of oxygen, were associated with PE. The area under the receiver operating characteristic curve of the model was 0.750 [95% confidence interval (CI): 0.721–0.783] in the training cohort and 0.742 (95% CI: 0.689–0.787) in the validation cohort, indicating that the model showed a good differential performance. A good consistency between the prediction and the real observation was presented in the training and validation cohorts. The decision curve analysis in the training and validation cohorts showed that the numerical model had a good net clinical benefit.

**Conclusion:**

We established a novel numerical model to predict the risk factors for PE in neurology department suspected PE patients. Our findings may help doctors to develop individualized treatment plans and PE prevention strategies.

## Introduction

Pulmonary embolism (PE) is a fatal cardiovascular disorder that remains a challenge to doctors during clinical diagnosis and treatment. Approximately 300,000 people die from PE every year in the United States of America, which ranks PE high among the causes of cardio-cerebrovascular mortality (1). This is especially true in elderly patients as it is hard to distinguish their symptoms of PE from other mild illnesses (2). The proportion of elderly patients in neurology departments cannot be neglected; the characteristics of these neurology department patients include aging, being bedridden for a long term, and presenting many concomitant diseases. These elderly patients are prone to lower extremity deep vein thrombosis and to develop PE (3). The presence of PE is a risk factor for stroke, cerebral infraction, and transient ischemic attack (4–6). Furthermore, PE-related death accounts for 20–25% of early deaths in stroke patients (7). Therefore, timely and accurate diagnosis of PE is crucial for the prognosis of neurology department patients.

Computed tomography pulmonary angiography (CTPA) is recommended for the diagnosis and risk-level assessment of PE (8–10). However, CTPA is time-consuming and expensive and can even cause serious side effects in patients. Therefore, it would be convenient to have a simple and fast risk prediction model to predict the probability of PE occurrence. Many researchers had created a variety of risk assessment models (RAMs) to predict PE, and their usability has been continuously validated. Robert-Ebadi et al. verified the feasibility of the simplified Geneva score in the clinic in 2017 (11). Freund et al. explored the safety of the PE rule-out criteria with a randomized clinical trial in 2018 (12). In addition, in 2019, van der Pol et al. assessed whether a pregnancy-adapted algorithm could help pregnant women avoid the imaging diagnosis for safety reasons (13). Furthermore, Kirsch et al. (14) demonstrated the ability of the Wells score to predict PE, which indicated that a Wells score above 4 was associated with PE; however, the performance of the Wells score was unreliable.

There have been many debates regarding the use of these RAMs; however, there are no consensual methods to diagnose PE. Currently, a RAM specifically for use with neurology department patients has not been developed. In recent years, we published a number of articles related to PE (15–17), and on this basis, we wanted to develop a numerical model that could rapidly determine the risk of PE in neurology department patients.

The numerical model (18, 19) is a graphical description of data, which presents the regression model in an accessible way, thus simplifying the risk assessment, providing a user-friendly interface for medical practitioners to map the probability of events to a single patient, and enhancing the clinical decision-making of doctors and patients. Therefore, the purpose of this study was to develop and validate a numerical model for the prediction of the risk of PE in neurology department suspected PE patients.

## Materials and methods

### Study population

Neurology department patients with suspected PE who were admitted at the Affiliated Dongyang Hospital of the Wenzhou Medical University from January 2012 to December 2021 were considered for enrollment in our study. The patients who had undergone CTPA examination were suspected PE. The subjects' data were retrospectively collected from our clinical research data platform. After baseline data clearance and extraction, the medical records of 1,578 subjects were included in the statistical analysis. Subjects were randomly divided into the training cohort and the validation cohort at a ratio of 7:3.

Ethical approval for this retrospective study was obtained from the Medical Ethics Committee of the Affiliated Dongyang Hospital of the Wenzhou Medical University (No.: 2022-YX-160), and the requirement for informed consent was waived as the medical information of all patients was anonymized and de-identified prior to conducting the analysis. Our study was conducted in accordance with the Declaration of Helsinki.

### Study outcomes and data collection

**Figure 1:**
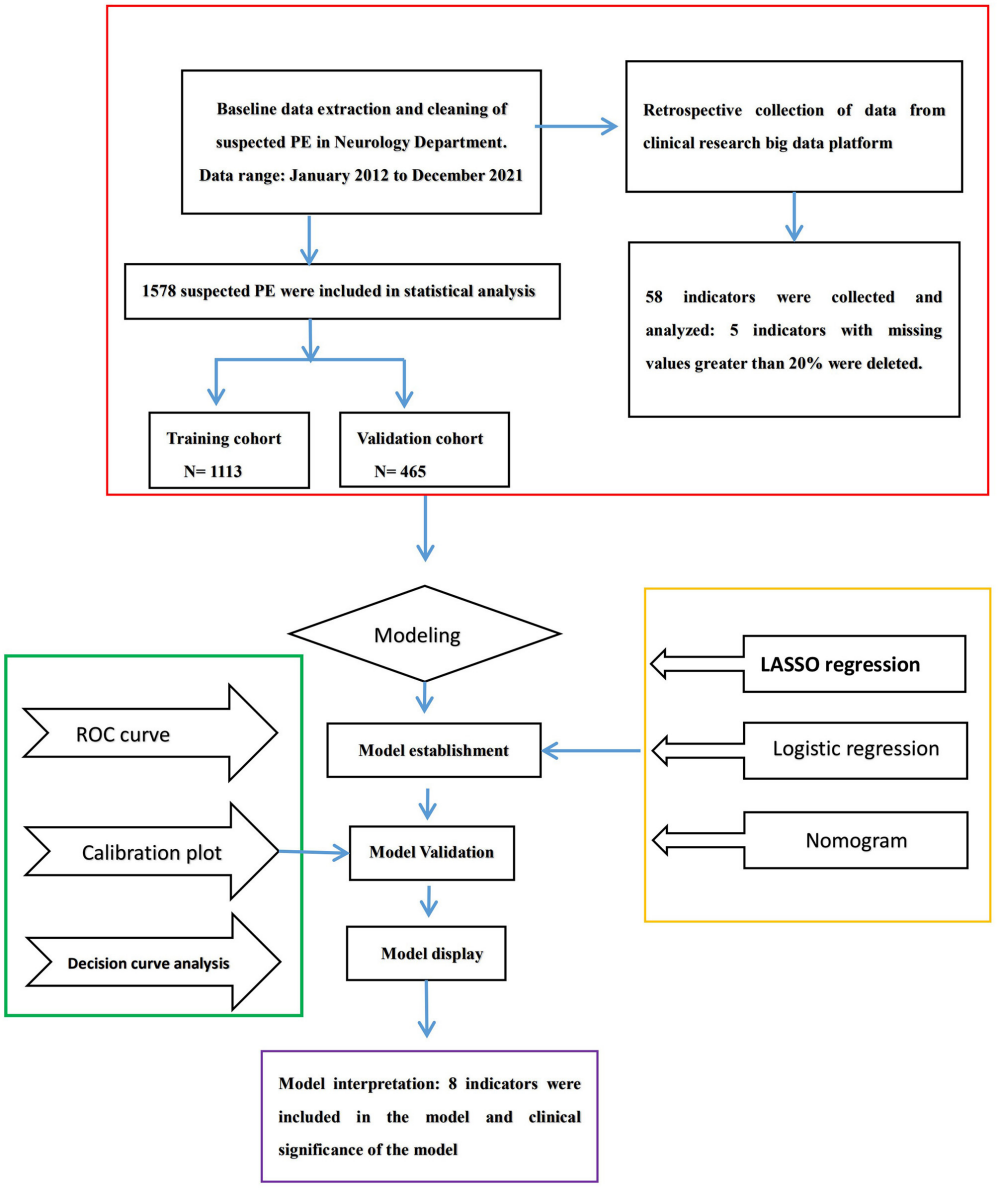
Flowchart of the processing step for predicting PE.

Pulmonary embolism (PE) was defined in accordance with the criteria of the European Society of Cardiology Guidelines (20). The diagnosis of PE was based on a filling defect of the pulmonary artery system (including the subsegment pulmonary artery) in computed tomography pulmonary angiography (CTPA). The past medical history, complications, individual clinical features, and clinical biomarker data were collected. The indicators we chose, for example, blood oxygen saturation, systolic blood pressure, and diastolic pressure, were strictly defined from admission to CTPA, and the lowest result was selected; for other indicators, the highest result was selected. Our research flowchart is shown in Figure 1.

### Statistical analysis

Data were statistically analyzed by RStudio software for Windows. Categorical variables were expressed as frequency with percentages and were compared using the chi-square (χ^2^) test or Fisher's exact test. Continuous variables were expressed as the mean with standard deviation (SD) or median and interquartile range (IQR), and were compared using the Student's *t*-test or the Mann–Whitney *U*-test. All subjects contained 58 variables. To guarantee the reliability of the data, five indicators with missing values >20% were deleted. The “mice” package in R software for multiple imputation techniques was used (21) to impute the remaining missing predictor values. The “glmnet” package for the least absolute shrinkage and selection operator regression (LASSO) analysis was used to select the optimal predictive features, and an “rms” package for multivariate logistic regression analysis was used to establish the numerical model (22–24). The “regplot” package in R software was used for the nomogram. The features were presented as the odds ratio (OR) and 95% confidence interval (CI). A two-sided *p*-value of < 0.05 was considered to statistically significant.

### Model development, validation, and evaluation

The least absolute shrinkage and selection operator regression analysis was used to select the optimal predictive features, and a multivariable logistic regression analysis was used to establish a numerical model to predict PE in the training cohort. The model performance was assessed and validated for discrimination, calibration, and clinical utility in both cohorts (25). The differentiation in the model was evaluated using the “pROC” package for the area under the receiver operating characteristic (ROC) curve (AUC), the “calibrate” package for calibration curve analysis to evaluate the calibration of the model, and the “rmda” package for decision curve analysis (DCA), which were used to quantify the net benefit under different threshold probabilities to determine the clinical utility of the model.

## Results

### Characteristics of the study population

After excluding five variables with missing information in more than 20% of patients, we involved 53 variables with missing data in < 20% of patients involved in this study (shown in Supplementary Figure 1). The missing data for the 53 variables ranged from 0.00 to 18.69%; thus, the multiple imputation technique was used to impute the missing data. A total of 1,578 subjects with suspected PE were enrolled in this study. The incidence of PE in our study was 24.33%. The baseline characteristics of neurology department patients with suspected PE are displayed in Table 1. We divided the patients into the training cohort (1,113 patients) and the validation cohort (465 patients). Basic characteristics of the patients in the training and validation cohorts are presented in Table 2.

**Table 1:** Baseline characteristics of subjects.

**Variables**	**Total (*n* = 1,578)**	**No PE (*n* = 1,194)**	**PE (*n* = 384)**	***p***
Gender, *n* (%)				0.114
Women	822 (52.1)	608 (50.9)	214 (55.7)	
Men	756 (47.9)	586 (49.1)	170 (44.3)	
Age (years), median (Q1, Q3)	75 (65, 81)	73 (63, 81)	78 (71, 83)	< 0.001
Breathing (/min), median (Q1, Q3)	22 (20, 25)	22 (20, 24)	23 (20, 28)	< 0.001
Pulse (/min), median (Q1, Q3)	98 (88, 111)	96 (86, 108)	103.5 (96, 118.25)	< 0.001
Systolic pressure (mmHg), median (Q1, Q3)	99 (92, 107)	100 (92, 109)	96 (91, 104)	< 0.001
Diastolic pressure (mmHg), median (Q1, Q3)	52 (46, 58)	52 (47, 59)	50 (45, 54)	< 0.001
Headache, *n* (%)				0.914
No	1,471 (93.2)	1,114 (93.3)	357 (93)	
Yes	107 (6.8)	80 (6.7)	27 (7)	
Dizzy, *n* (%)				0.069
No	1,400 (88.7)	1,049 (87.9)	351 (91.4)	
Yes	178 (11.3)	145 (12.1)	33 (8.6)	
Chest tightness, *n* (%)				0.502
No	1,354 (85.8)	1,029 (86.2)	325 (84.6)	
Yes	224 (14.2)	165 (13.8)	59 (15.4)	
Anhelation, *n* (%)				0.086
No	1,467 (93)	1,118 (93.6)	349 (90.9)	
Yes	111 (7)	76 (6.4)	35 (9.1)	
Hemoptysis, *n* (%)				0.159
No	1,572 (99.6)	1,191 (99.7)	381 (99.2)	
Yes	6 (0.4)	3 (0.3)	3 (0.8)	
Chest pain, *n* (%)				1
No	1,558 (98.7)	1,179 (98.7)	379 (98.7)	
Yes	20 (1.3)	15 (1.3)	5 (1.3)	
Syncope, *n* (%)				< 0.001
No	1,359 (86.1)	995 (83.3)	364 (94.8)	
Yes	219 (13.9)	199 (16.7)	20 (5.2)	
Cough, *n* (%)				0.114
No	1,321 (83.7)	1,010 (84.6)	311 (81)	
Yes	257 (16.3)	184 (15.4)	73 (19)	
Fever, *n* (%)				0.62
No	1,542 (97.7)	1,165 (97.6)	377 (98.2)	
Yes	36 (2.3)	29 (2.4)	7 (1.8)	
Lower limb edema, *n* (%)				0.932
No	1,548 (98.1)	1,172 (98.2)	376 (97.9)	
Yes	30 (1.9)	22 (1.8)	8 (2.1)	
COPD, *n* (%)				0.022
No	1,349 (85.5)	1,035 (86.7)	314 (81.8)	
Yes	229 (14.5)	159 (13.3)	70 (18.2)	
Hypertension, *n* (%)				0.111
No	561 (35.6)	438 (36.7)	123 (32)	
Yes	1,017 (64.4)	756 (63.3)	261 (68)	
Diabetes, *n* (%)				0.173
No	1,297 (82.2)	972 (81.4)	325 (84.6)	
Yes	281 (17.8)	222 (18.6)	59 (15.4)	
Coronary heart disease, *n* (%)				0.133
No	1,241 (78.6)	950 (79.6)	291 (75.8)	
Yes	337 (21.4)	244 (20.4)	93 (24.2)	
Hyperlipidemia, *n* (%)				1
No	1,525 (96.6)	1,154 (96.6)	371 (96.6)	
Yes	53 (3.4)	40 (3.4)	13 (3.4)	
Atrial fibrillation, *n* (%)				< 0.001
No	1,435 (90.9)	1,110 (93)	325 (84.6)	
Yes	143 (9.1)	84 (7)	59 (15.4)	
Operation, *n* (%)				0.149
No	1,567 (99.3)	1,188 (99.5)	379 (98.7)	
Yes	11 (0.7)	6 (0.5)	5 (1.3)	
Tumor, *n* (%)				0.484
No	1,477 (93.6)	1,121 (93.9)	356 (92.7)	
Yes	101 (6.4)	73 (6.1)	28 (7.3)	
Smoke, *n* (%)				0.179
No	1,152 (73)	861 (72.1)	291 (75.8)	
Yes	426 (27)	333 (27.9)	93 (24.2)	
Drink, *n* (%)				0.144
No	1,051 (66.6)	783 (65.6)	268 (69.8)	
Yes	527 (33.4)	411 (34.4)	116 (30.2)	
WBC (10^9^/L), median (Q1, Q3)	7.35 (5.65, 9.77)	7.1 (5.52, 9.49)	8.14 (6.42, 10.68)	< 0.001
Lactate (mmol/L), median (Q1, Q3)	1.5 (1.2, 2.07)	1.5 (1.2, 2)	1.55 (1.2, 2.1)	0.523
RBC (10^12^/L), median (Q1, Q3)	4.32 (4, 4.68)	4.33 (4, 4.68)	4.32 (4.01, 4.65)	0.918
Mg (mmol/L), median (Q1, Q3)	0.9 (0.84, 0.96)	0.9 (0.85, 0.96)	0.89 (0.84, 0.95)	0.011
HGB (g/L), median (Q1, Q3)	133 (122, 143)	132 (121.25, 143)	134 (123, 143)	0.212
Hct, median (Q1, Q3)	0.4 (0.37, 0.43)	0.4 (0.37, 0.43)	0.41 (0.38, 0.43)	0.131
Neutrophil percent, median (Q1, Q3)	0.73 (0.64, 0.83)	0.71 (0.62, 0.82)	0.78 (0.69, 0.86)	< 0.001
Neutrophil count(10^9^/L), median (Q1, Q3)	5.11 (3.63, 7.72)	4.84 (3.47, 7.38)	6.18 (4.38, 8.55)	< 0.001
Lymphocyte percent, median (Q1, Q3)	0.27 (0.2, 0.34)	0.27 (0.2, 0.34)	0.25 (0.19, 0.32)	< 0.001
Lymphocyte count (10^9^/L), median (Q1, Q3)	1.58 (1.25, 2.03)	1.6 (1.27, 2.05)	1.53 (1.21, 2)	0.109
PLT (10^9^/L), median (Q1, Q3)	211 (174, 254.75)	211 (175, 252.75)	211.5 (170, 260.25)	0.642
ALB (g/L), median (Q1, Q3)	37.7 (35.2, 40.2)	37.9 (35.62, 40.4)	37 (34.7, 39.4)	< 0.001
PDW (%), median (Q1, Q3)	16 (13.8, 16.4)	15.9 (13.4, 16.3)	16.1 (15.6, 16.4)	< 0.001
RDW (%), median (Q1, Q3)	0.13 (0.12, 0.14)	0.13 (0.12, 0.14)	0.13 (0.13, 0.14)	0.46
HDL (mmol/L), median (Q1, Q3)	1.08 (0.91, 1.3)	1.07 (0.9, 1.29)	1.11 (0.96, 1.32)	0.009
LDL (mmol/L), median (Q1, Q3)	2.45 (1.92, 3.03)	2.4 (1.9, 2.97)	2.6 (2, 3.17)	0.001
Apolipoprotein A1 (g/L), median (Q1, Q3)	1.11 (0.93, 1.34)	1.1 (0.93, 1.33)	1.11 (0.94, 1.38)	0.235
Apolipoprotein B(g/L), median (Q1, Q3)	0.85 (0.69, 1.02)	0.84 (0.69, 1.01)	0.88 (0.71, 1.05)	0.061
TG(mmol/L), median (Q1, Q3)	1.28 (0.98, 1.76)	1.29 (1, 1.8)	1.21 (0.93, 1.56)	0.002
TC (mmol/L), median (Q1, Q3)	4.22 (3.62, 4.93)	4.2 (3.6, 4.86)	4.37 (3.7, 5.04)	0.033
Fibrinogen (g/L), median (Q1, Q3)	3.64 (2.99, 4.54)	3.58 (2.97, 4.48)	3.83 (3.07, 4.67)	0.024
D-Dimer (mg/L), median (Q1, Q3)	1.62 (0.78, 4.99)	1.19 (0.69, 3.59)	4.3 (2.07, 8.27)	< 0.001
PT(s), median (Q1, Q3)	13.6 (13, 14.3)	13.5 (13, 14.2)	13.9 (13.3, 14.5)	< 0.001
APTT(s), median (Q1, Q3)	37.2 (34.6, 40.3)	37 (34.5, 40.1)	37.65 (35, 40.82)	0.038
TT(s), median (Q1, Q3)	16.5 (15.9, 17.1)	16.5 (15.9, 17)	16.5 (15.9, 17.1)	0.853
PO_2_ (mmHg), median (Q1, Q3)	73.3 (64.4, 86)	74.7 (65.22, 89.7)	69.6 (62.7, 78.43)	< 0.001
FIO_2_ (%), median (Q1, Q3)	29 (21, 33)	21 (21, 33)	29 (21, 33)	0.131

WBC, white blood cell count; RBC, red blood cell count; Mg, magnesium; HGB, hemoglobin; Hct, hematocrit; PLT, platelet count; ALB, albumin; PDW, platelet distribution width; RDW, red blood cell distribution width; HDL, high-density lipoprotein; LDL, low-density lipoprotein; TG, triglyceride; TC, total cholesterol; PT, prothrombin time; APTT, activated partial prothrombin time; TT, thrombin time; PO_2_, arterial partial pressure of oxygen; and FIO_2_, oxygen concentration fraction in the inhaled gas.

**Table 2:** The baseline characteristics of the enrolled patients in the training and validation cohorts.

**Variables**	**Total**	**Training**	**Validation**	***p***

	**(*****n*** = **1,578)**	**(*****n*** = **1,113)**	**(*****n*** = **465)**	
PE, *n* (%)				0.576
No	1,194 (75.7)	847 (76.1)	347 (74.6)	
Yes	384 (24.3)	266 (23.9)	118 (25.4)	
Gender, *n* (%)				1
Women	822 (52.1)	580 (52.1)	242 (52)	
Men	756 (47.9)	533 (47.9)	223 (48)	
Age (years), median (Q1, Q3)	75 (65, 81)	74 (65, 81)	76 (66, 82)	0.156
Breathing (/min), median (Q1, Q3)	22 (20, 25)	22 (20, 25)	22 (20, 25)	0.954
Pulse (/min), median (Q1, Q3)	98 (88, 111)	98 (88, 112)	98 (88, 110)	0.899
Systolic pressure (mmHg), median (Q1, Q3)	99 (92, 107)	99 (92, 108)	98 (92, 106)	0.208
Diastolic pressure (mmHg), median (Q1, Q3)	52 (46, 58)	52 (47, 58)	51 (46, 58)	0.219
Headache, *n* (%)				0.506
No	1,471 (93.2)	1,034 (92.9)	437 (94)	
Yes	107 (6.8)	79 (7.1)	28 (6)	
Dizzy, *n* (%)				0.721
No	1,400 (88.7)	990 (88.9)	410 (88.2)	
Yes	178 (11.3)	123 (11.1)	55 (11.8)	
Chest tightness, *n* (%)				0.813
No	1,354 (85.8)	957 (86)	397 (85.4)	
Yes	224 (14.2)	156 (14)	68 (14.6)	
Anhelation, *n* (%)				0.413
No	1,467 (93)	1,039 (93.4)	428 (92)	
Yes	111 (7)	74 (6.6)	37 (8)	
Hemoptysis, *n* (%)				1
No	1,572 (99.6)	1,109 (99.6)	463 (99.6)	
Yes	6 (0.4)	4 (0.4)	2 (0.4)	
Chest pain, *n* (%)				0.492
No	1,558 (98.7)	1,097 (98.6)	461 (99.1)	
Yes	20 (1.3)	16 (1.4)	4 (0.9)	
Syncope, *n* (%)				0.745
No	1,359 (86.1)	956 (85.9)	403 (86.7)	
Yes	219 (13.9)	157 (14.1)	62 (13.3)	
Cough, *n* (%)				0.573
No	1,321 (83.7)	936 (84.1)	385 (82.8)	
Yes	257 (16.3)	177 (15.9)	80 (17.2)	
Fever, *n* (%)				0.682
No	1,542 (97.7)	1,086 (97.6)	456 (98.1)	
Yes	36 (2.3)	27 (2.4)	9 (1.9)	
Lower limb edema, *n* (%)				0.344
No	1,548 (98.1)	1,089 (97.8)	459 (98.7)	
Yes	30 (1.9)	24 (2.2)	6 (1.3)	
COPD, *n* (%)				0.533
No	1,349 (85.5)	947 (85.1)	402 (86.5)	
Yes	229 (14.5)	166 (14.9)	63 (13.5)	
Hypertension, *n* (%)				0.983
No	561 (35.6)	395 (35.5)	166 (35.7)	
Yes	1,017 (64.4)	718 (64.5)	299 (64.3)	
Diabetes, *n* (%)				0.739
No	1,297 (82.2)	912 (81.9)	385 (82.8)	
Yes	281 (17.8)	201 (18.1)	80 (17.2)	
Coronary heart disease, *n* (%)				0.608
No	1,241 (78.6)	871 (78.3)	370 (79.6)	
Yes	337 (21.4)	242 (21.7)	95 (20.4)	
Hyperlipidemia, *n* (%)				0.135
No	1,525 (96.6)	1,081 (97.1)	444 (95.5)	
Yes	53 (3.4)	32 (2.9)	21 (4.5)	
Atrial fibrillation, *n* (%)				0.278
No	1,435 (90.9)	1,006 (90.4)	429 (92.3)	
Yes	143 (9.1)	107 (9.6)	36 (7.7)	
Operation, *n* (%)				1
No	1,567 (99.3)	1,105 (99.3)	462 (99.4)	
Yes	11 (0.7)	8 (0.7)	3 (0.6)	
Tumor, *n* (%)				0.776
No	1,477 (93.6)	1,040 (93.4)	437 (94)	
Yes	101 (6.4)	73 (6.6)	28 (6)	
Smoke, *n* (%)				0.453
No	1,152 (73)	806 (72.4)	346 (74.4)	
Yes	426 (27)	307 (27.6)	119 (25.6)	
Drink, *n* (%)				0.707
No	1,051 (66.6)	745 (66.9)	306 (65.8)	
Yes	527 (33.4)	368 (33.1)	159 (34.2)	
WBC (10^9^/L), median (Q1, Q3)	7.35 (5.65, 9.77)	7.35 (5.66, 9.78)	7.3 (5.6, 9.69)	0.754
Lactate (mmol/), median (Q1, Q3)	1.5 (1.2, 2.07)	1.5 (1.2, 2)	1.5 (1.1, 2.1)	0.606
RBC (10^12^/L), median (Q1, Q3)	4.32 (4, 4.68)	4.33 (4, 4.67)	4.3 (4, 4.69)	0.906
Mg (mmol/L), median (Q1, Q3)	0.9 (0.84, 0.96)	0.9 (0.85, 0.96)	0.9 (0.84, 0.96)	0.809
HGB (g/L), median (Q1, Q3)	133 (122, 143)	133 (122, 143)	132 (122, 143)	0.758
Hct, median (Q1, Q3)	0.4 (0.37, 0.43)	0.4 (0.37, 0.43)	0.4 (0.37, 0.43)	0.892
Neutrophil percent, median (Q1, Q3)	0.73 (0.64, 0.83)	0.73 (0.64, 0.82)	0.74 (0.64, 0.84)	0.212
Neutrophil count (10^9^/L), median (Q1, Q3)	5.11 (3.63, 7.72)	5.11 (3.65, 7.66)	5.06 (3.55, 7.83)	0.92
Lymphocyte percent, median (Q1, Q3)	0.27 (0.2, 0.34)	0.27 (0.2, 0.34)	0.27 (0.2, 0.34)	0.78
Lymphocyte count (10^9^/L), median (Q1, Q3)	1.58 (1.25, 2.03)	1.58 (1.25, 2.05)	1.57 (1.25, 2.01)	0.427
PLT (10^9^/L), median (Q1, Q3)	211 (174, 254.75)	212 (175, 255)	208 (169, 253)	0.097
ALB (g/L), median (Q1, Q3)	37.7 (35.2, 40.2)	37.7 (35.3, 40.3)	37.8 (35.1, 40.1)	0.396
PDW (%), median (Q1, Q3)	16 (13.8, 16.4)	15.9 (13.8, 16.4)	16 (13.7, 16.3)	0.93
RDW (%), median (Q1, Q3)	0.13 (0.12, 0.14)	0.13 (0.12, 0.14)	0.13 (0.13, 0.14)	0.842
HDL (mmol/L), median (Q1, Q3)	1.08 (0.91, 1.3)	1.09 (0.91, 1.31)	1.06 (0.9, 1.28)	0.155
LDL (mmol/L), median (Q1, Q3)	2.45 (1.92, 3.03)	2.46 (1.94, 3.02)	2.39 (1.81, 3.04)	0.166
Apolipoprotein A1 (g/L), median (Q1, Q3)	1.11 (0.93, 1.34)	1.11 (0.93, 1.35)	1.09 (0.92, 1.31)	0.098
Apolipoprotein B(g/L), median (Q1, Q3)	0.85 (0.69, 1.02)	0.85 (0.69, 1.02)	0.85 (0.69, 1.01)	0.51
TG (mmol/L), median (Q1, Q3)	1.28 (0.98, 1.76)	1.28 (0.99, 1.76)	1.25 (0.95, 1.74)	0.382
TC (mmol/L), median (Q1, Q3)	4.22 (3.62, 4.93)	4.25 (3.65, 4.93)	4.2 (3.58, 4.91)	0.29
Fibrinogen (g/L), median (Q1, Q3)	3.64 (2.99, 4.54)	3.63 (3.01, 4.5)	3.65 (2.96, 4.72)	0.507
D-Dimer (mg/L), median (Q1, Q3)	1.62 (0.78, 4.99)	1.5 (0.76, 4.65)	1.94 (0.83, 5.4)	0.081
PT(s), median (Q1, Q3)	13.6 (13, 14.3)	13.6 (13, 14.3)	13.7 (13.1, 14.3)	0.027
APTT(s), median (Q1, Q3)	37.2 (34.6, 40.3)	37.1 (34.6, 40.2)	37.6 (34.8, 40.6)	0.177
TT(s), median (Q1, Q3)	16.5 (15.9, 17.1)	16.5 (15.9, 17.1)	16.4 (15.9, 17)	0.159
PO_2_ (mmHg), median (Q1, Q3)	73.3 (64.4, 86)	72.9 (64.3, 85.5)	73.6 (64.6, 88.3)	0.482
FIO_2_ (%), median (Q1, Q3)	29 (21, 33)	29 (21, 33)	21 (21, 33)	0.541

WBC, white blood cell count; RBC, red blood cell count; Mg, magnesium; HGB, hemoglobin; Hct, hematocrit; PLT, platelet count; ALB, albumin; PDW, platelet distribution width; RDW, red blood cell distribution width; HDL, high-density lipoprotein; LDL, low-density lipoprotein; TG, triglyceride; TC, total cholesterol; PT, prothrombin time; APTT, activated partial prothrombin time; TT, thrombin time; PO_2_, arterial partial pressure of oxygen; and FIO_2_, oxygen concentration fraction in the inhaled gas.

### Selected predictors

**Figure 2:**
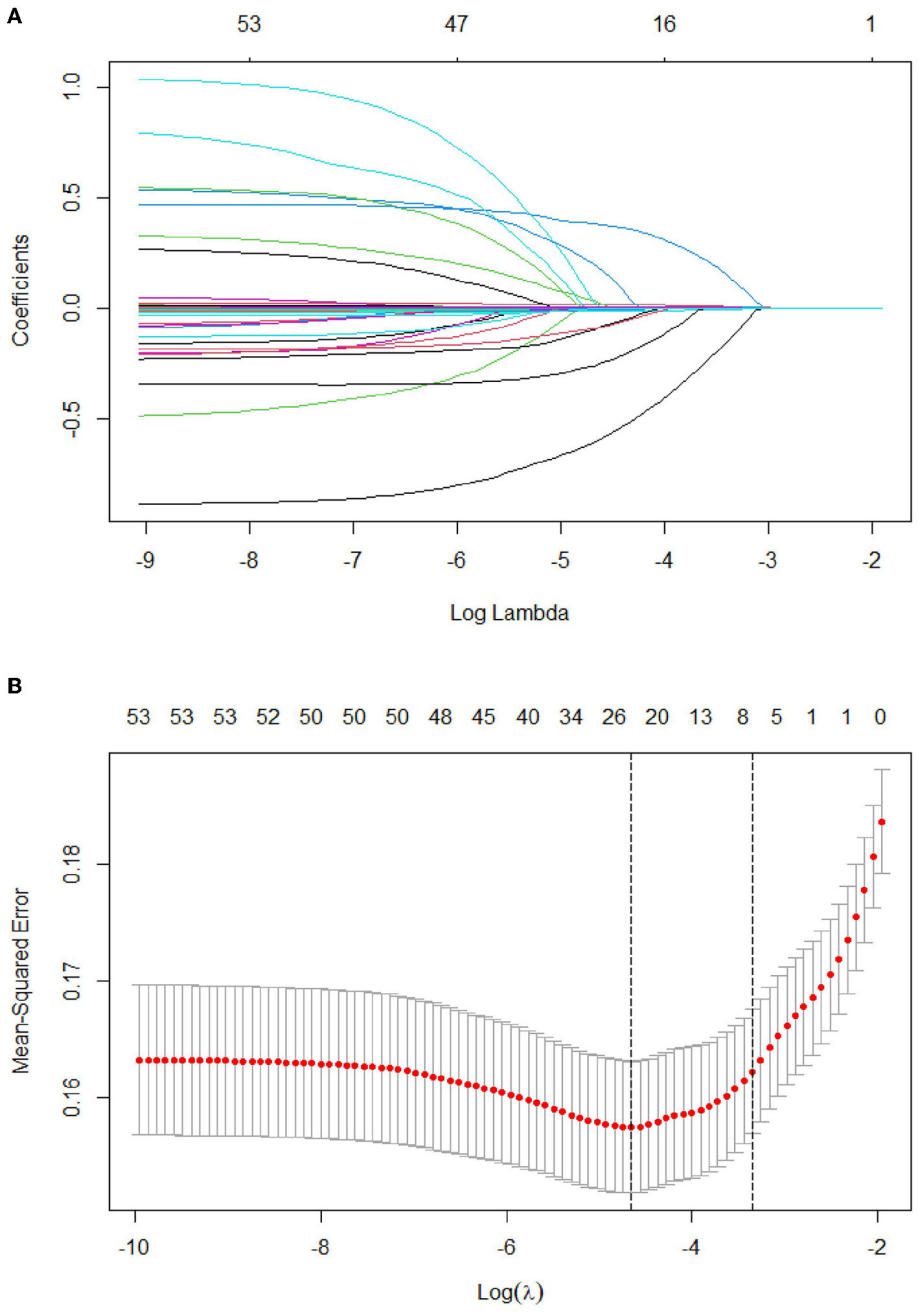
Tuning parameter selection using the LASSO regression in the training cohort. **(A)** LASSO coefficient profiles of the clinical features. **(B)** The optimal penalization coefficient lambda was generated in the LASSO *via* 10-fold cross-validation. The lambda value of the 1-fold mean square error for the training cohort was given.

Of the 53 variables, eight potential predictive features were finally selected based on the LASSO regression analysis (Figures 2A, B). The optimal predictors included age, pulse, systolic pressure, hemoglobin (HGB), neutrophil count (N), low-density lipoprotein (LDL), D-dimer (DD), and partial pressure of oxygen (PO_2_). The eight potential predictive features screened from the LASSO regression analysis were used to create the final model based on the multivariable logistic regression analysis in the training cohort (Table 3). The prevalence of PE is 23.9% in the training and 25.4% in the validation cohorts (Table 2). The sensitivity of our model is 94.17%, the specificity is 17.56%, the positive predictive value is 77.47%, and the negative predictive value is 50% in the training cohort.

**Table 3:** Final model coefficients.

**Variables**	**β**	**SE**	**OR**	**95% CI**	***P***
Age	0.039	0.008	1.04	1.02–1.06	< 0.001
Pulse	0.006	0.004	1.01	1–1.01	0.135
Systolic_pressure	−0.019	0.006	0.98	0.97–0.99	0.002
HGB	0.009	0.005	1.01	1–1.02	0.05
Neutrophil count	0.016	0.022	1.02	0.97–1.06	0.462
LDL	0.219	0.092	1.24	1.04–1.49	0.018
D-Dimer	0.093	0.015	1.1	1.07–1.13	< 0.001
PO_2_	−0.004	0.003	1	0.99-1	0.175

HGB, hemoglobin; LDL, low-density lipoprotein; and PO_2_,arterial partial pressure of oxygen.

### Construction and validation of the model

**Figure 3:**
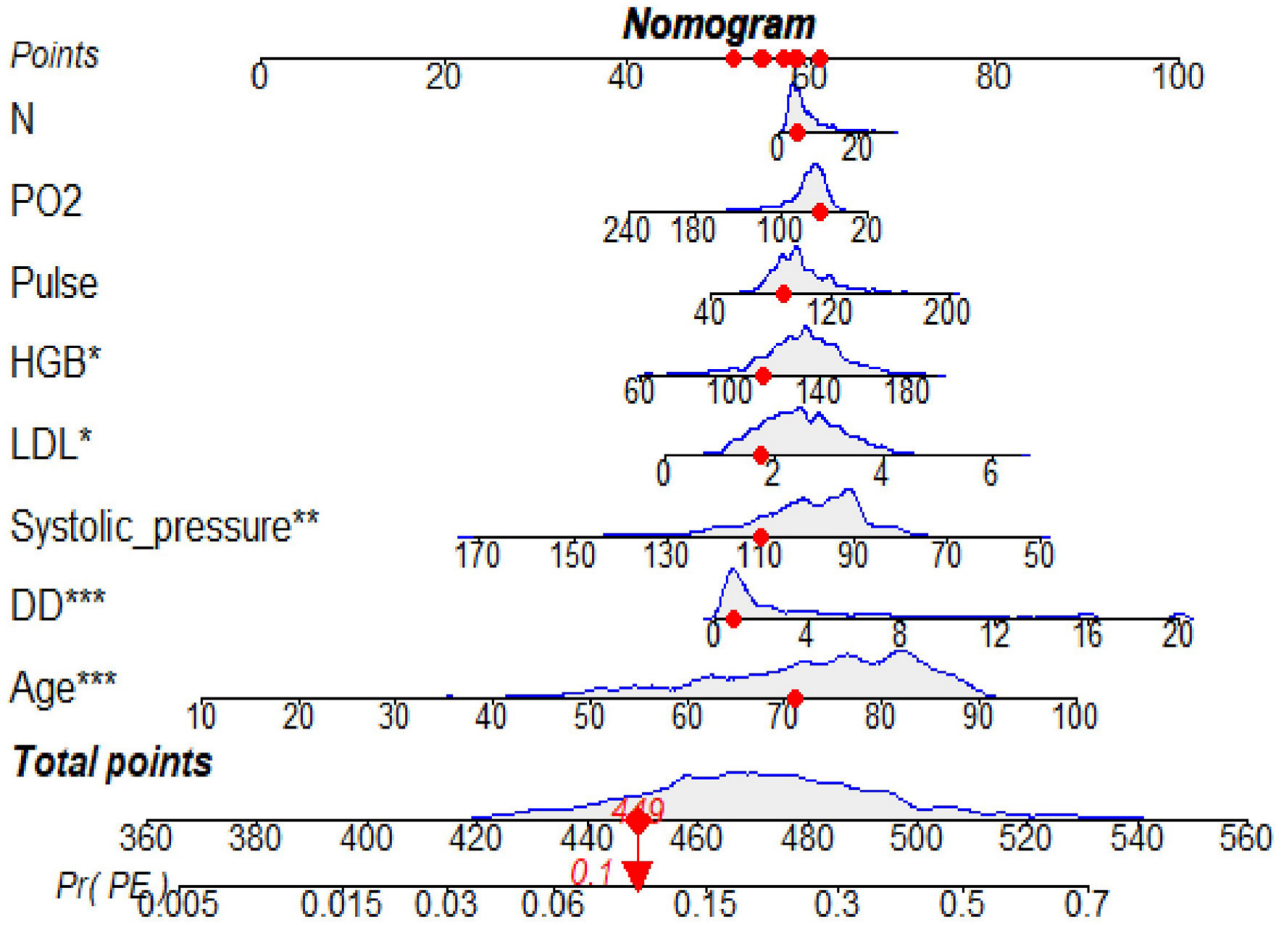
A nomogram based on the combination of eight indicators was developed using logistic regression analysis. If the neurology department patient had a total score of 449 points, this corresponded to an ~10% risk of PE and then the probability of the PE was 0.1 (red numbers). HGB, hemoglobin; *N*, neutrophil count; LDL, low-density lipoprotein; DD, D-dimer; PO2, oxygen partial pressure (PO_2_). *p < 0.05, **p < 0.01, ***p < 0.001.

**Figure 4:**
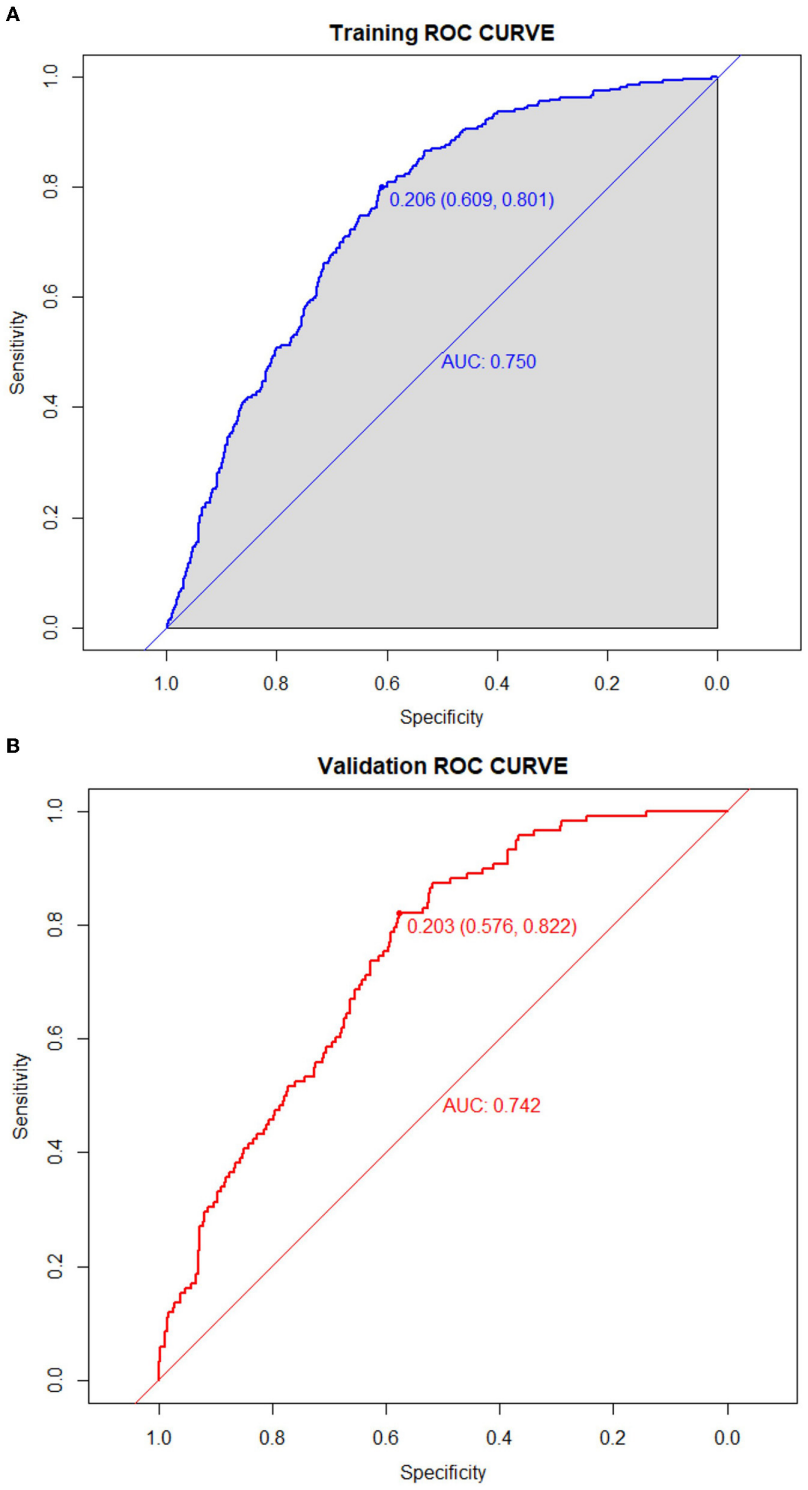
Receiver operating characteristic curves of the model distinguishing PE from non-PE in the training **(A)** and validation **(B)** cohorts.

**Figure 5:**
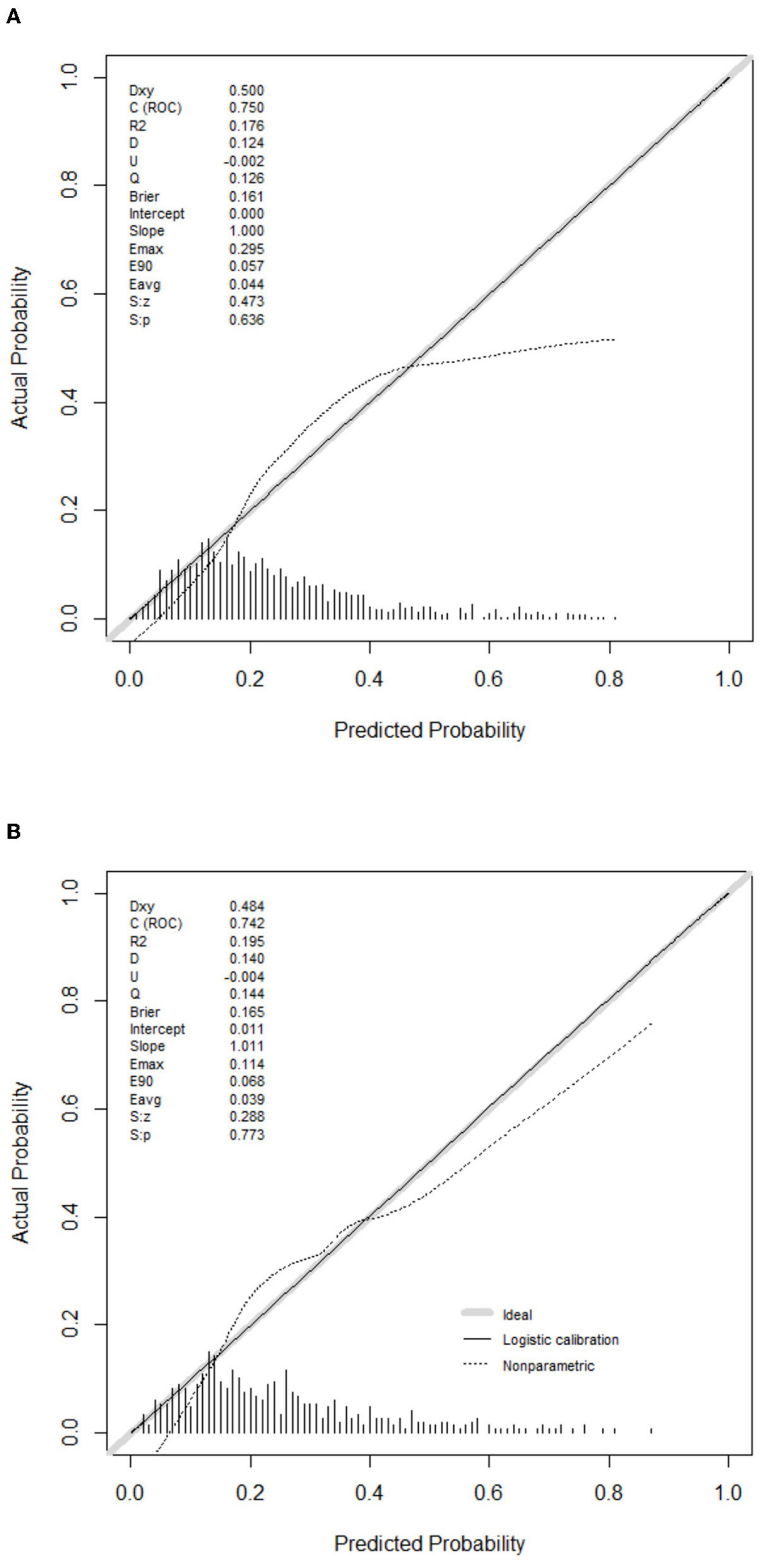
Calibration curves of the model in the training **(A)** and validation **(B)** cohorts.

**Figure 6:**
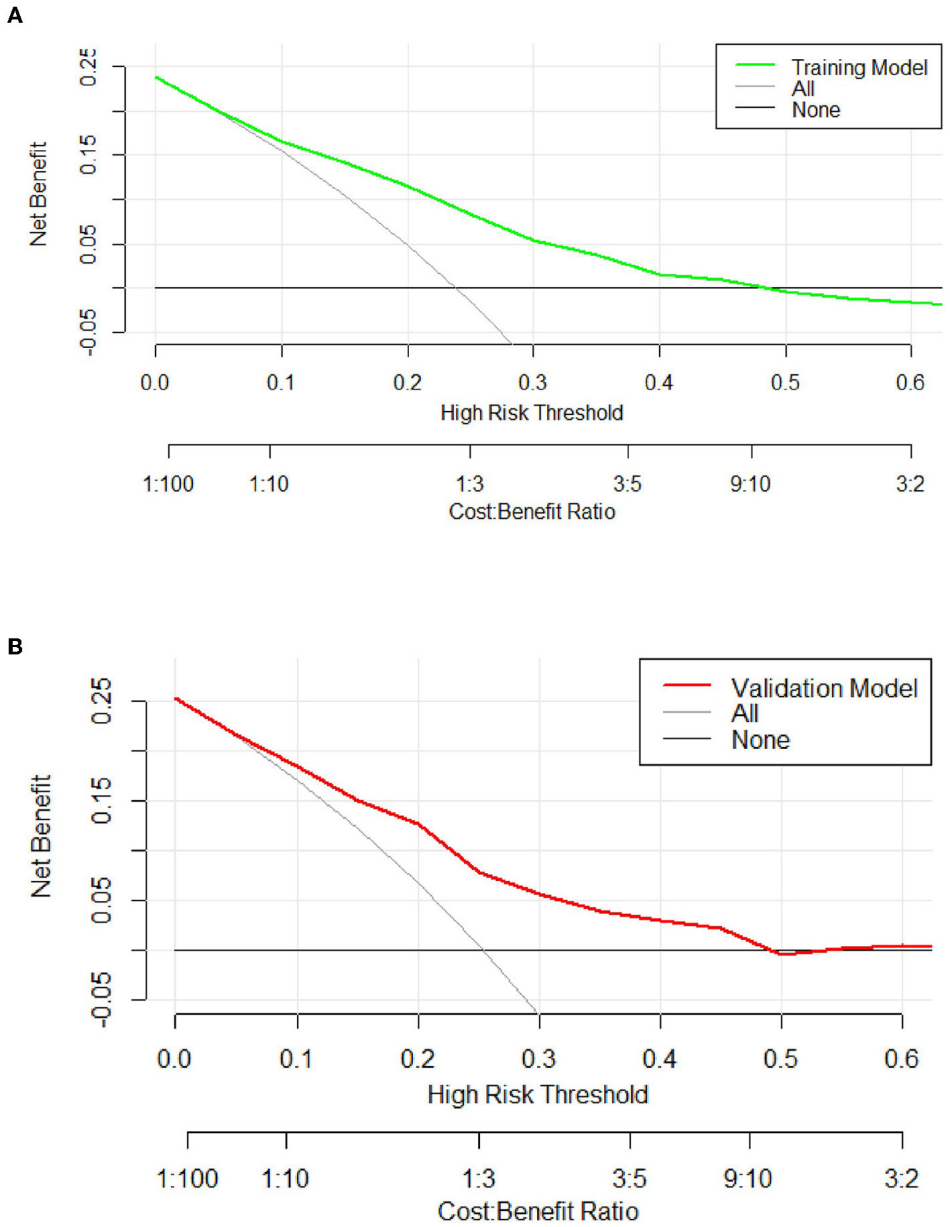
The decision curve of the model in the training **(A)** and validation **(B)** cohort. If the risk threshold is < 50%, the nomogram model will obtain more benefit than all treatments (assuming that all neurology department patients were PE) or no treatment (assuming all neurology department patients were non-PE).

The predictive model for PE was visualized by a nomogram in the training cohort, which is shown in Figure 3. Model discrimination, as quantified by the AUC, was 0.750 (95% CI: 0.721–0.783) in the training cohort and 0.742 (95% CI: 0.689–0.787) in the validation cohort, indicating that this numerical model can successfully distinguish PE from non-PE (Figures 4A, B). The calibration plots in the training and validation cohorts are shown in Figures 5A, B, which demonstrate a good consistency between the prediction and the real observation. The DCA in the training and validation cohorts indicated that the numerical model had a good net clinical benefit (Figures 6A, B).

## Discussion

In this study, we developed and validated a simple model to determine the possible risk factors for PE based on a 10-year retrospective study in a comprehensive hospital in China. Our novel numerical model incorporated eight parameters, namely, age, pulse, systolic pressure, HGB, N, LDL, DD, and PO_2_. All parameters are readily available clinical features and biomarkers in routine health examinations. Notably, the ROC analysis showed that the AUC was 0.750 (95% CI: 0.721–0.783), indicating that our model displayed good discrimination and calibration. Furthermore, the DCA in the training and validation cohorts indicated that our model had a good net clinical benefit.

Our research found that DD (OR: 1.10; 95% CI: 1.07–1.13) is an independent predictive factor for the increased risk of PE. This result is in accordance with other observations (26, 27), which found that a high DD level was attributable to the possibility of developing PE. In terms of biomarkers, DD is the only biomarker currently used in routine practice for predicting PE; however, it is unlikely to have adequate specificity in neurology department patients for positivity. A large sample study from 2000 to 2015 showed increased hospitalization rates and the highest inpatient mortality due to PE in elderly patients (28). In addition, a retrospective study demonstrated an association between age and the severity of submassive PE stadium (29). Our model also showed that age (OR: 1.04; 95% CI: 1.02–1.06) is a high-risk factor for PE, which is similar to previous studies. In our model, most of the factors were positively associated with the risk of PE, whereas systolic blood pressure and PO_2_ were negatively associated. A previous study showed that low systolic pressure was connected with an increased risk of PE-related mortality (30, 31). In hemodynamically stable patients, a lower PO_2_ (< 8 kPa) was still associated with an elevated risk of mortality (32). These conclusions were consistent with those of our study. Our data also revealed that two clinical symptoms, including pulse and systolic pressure, were incorporated into the model to predict PE. Consistent with our result, a previous study (33) showed that pulse and systolic pressure were good predictors in a model for the prognosis of PE. Relevant studies (34–37) have also shown that inflammation and dyslipidemia are factors affecting PE, which is similar to the presence of inflammation and blood lipid indicators in our model. However, there are still some differences between the indicators included in our model and those included in the previous models (38). Three possible explanations for the discrepant results are as follows: (1) there are no such indicators in our clinical research data platform; (2) indicators with missing values >20% were deleted; and (3) indicators were not included in the model after the analysis.

This retrospective study suggested that a nomogram developed with clinical features and biomarkers to generate a personalized evaluation of PE risk in neurology department patients may distinguish patients at high risk of PE. For example, if the neurology department patient had a total score of 449 points, this corresponded to an ~10% risk of PE, and the probability of the PE was 0.1. Clinicians can use this simple numerical model to categorize the neurology department patients as PE-likely or PE-unlikely, thus reducing unnecessary CTPA examinations. This model may also be helpful to identify high-risk patients early, evaluate thrombosis, and implement active and individualized anticoagulation therapy.

This study is subject to certain limitations. In this retrospective study, five indicators (blood oxygen saturation, BMI, ejection fraction, troponin T, and brain natriuretic peptide precursor) with missing values > 20% were deleted. Moreover, the additional disadvantages of this study were the limited sample of participants and a lack of information on sufficient variables. Additionally, the data were collected as a single-center retrospective study.

In conclusion, we developed a novel numerical model for selecting the risk factors for PE in suspected-PE patients in a neurology department. Our findings may help decision-makers weigh the risk of PE and appropriately select PE prevention strategies. In the future, a large-scale prospective multicenter cohort study would help to form an improved and updated clinical decision-making system.

## Data availability statement

The raw data supporting the conclusions of this article will be made available by the authors, without undue reservation.

## Ethics statement

The studies involving human participants were reviewed and approved by the Medical Ethics Committee of Affiliated Dongyang Hospital of Wenzhou Medical University. Written informed consent for participation was not required for this study in accordance with the national legislation and the institutional requirements.

## Author contributions

WM conceived and designed the research strategy. QJ, JL, and WM wrote the manuscript text. QL, FL, MX, and LW collected the clinical data and participated in the writing of the manuscript. WM, MX, and LW contributed to the analysis and interpretation of the data. All authors contributed to this manuscript and approved the submitted version of the manuscript.
