# Supplementary figures and images for: A nomogram for predicting the risk of pulmonary embolism in neurology department suspected PE patients: A 10-year retrospective analysis
Source: Front Neurol. 2023 Apr 5;14:1139598. doi: 10.3389/fneur.2023.1139598 (PMC10113433; doi:10.3389/fneur.2023.1139598)

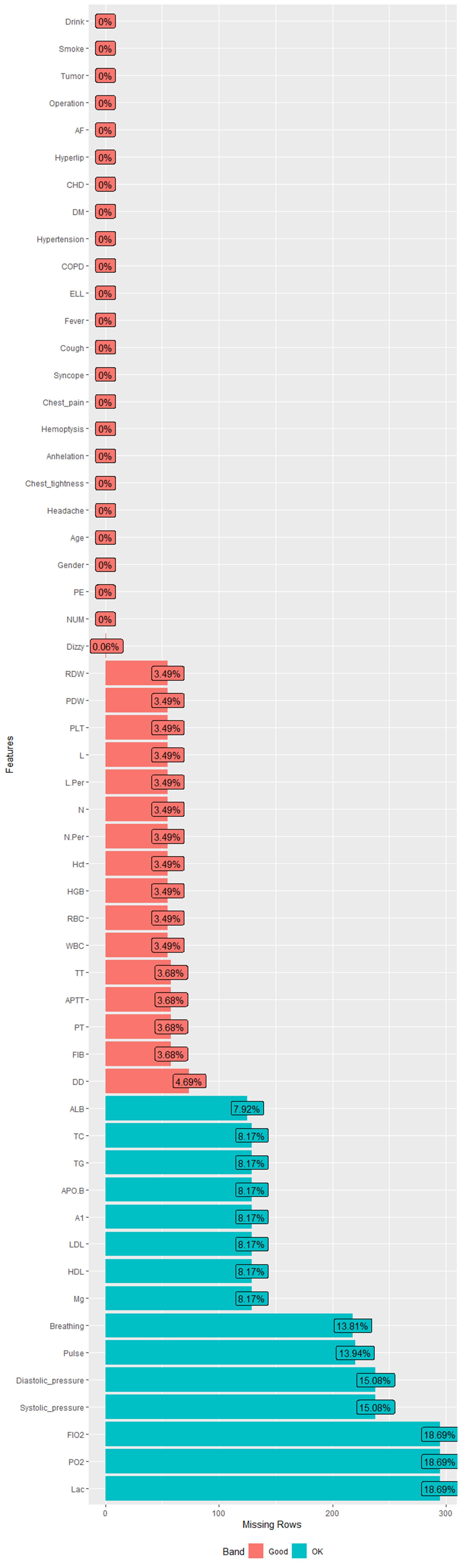

Supplement: Supplementary file 1 [file Image_1.TIFF]
